# Modeling Mongoose Rabies in the Caribbean: A Model-Guided Fieldwork Approach to Identify Research Priorities

**DOI:** 10.3390/v13020323

**Published:** 2021-02-20

**Authors:** Caroline C. Sauvé, Erin E. Rees, Amy T. Gilbert, Are R. Berentsen, Agathe Allibert, Patrick A. Leighton

**Affiliations:** 1 Epidemiology of Zoonoses and Public Health Research Group (GREZOSP), Faculty of Veterinary Medicine, Université de Montréal, 3190 Rue Sicotte, Saint-Hyacinthe, QC J2S 2M2, Canada; erin.rees@canada.ca (E.E.R.); agathe.allibert@umontreal.ca (A.A.); patrick.a.leighton@umontreal.ca (P.A.L.); 2 Centre de Recherche en Santé Publique, 7101 Avenue du Parc, Montréal, QC H3N 1X9, Canada; 3 National Microbiology Laboratory, Public Health Agency of Canada, 3190 Rue Sicotte, Saint-Hyacinthe, QC J2S 2M2, Canada; 4 National Wildlife Research Center, Wildlife Services, Animal and Plant Health Inspection Service, United Sates Department of Agriculture, 4101 LaPorte Avenue, Fort Collins, CO 80521, USA; amy.t.gilbert@usda.gov (A.T.G.); are.r.berentsen@usda.gov (A.R.B.)

**Keywords:** rabies, individual-based model, small Indian mongoose, *Urva auropunctata*, model-guided fieldwork, epidemiological model

## Abstract

We applied the model-guided fieldwork framework to the Caribbean mongoose rabies system by parametrizing a spatially-explicit, individual-based model, and by performing an uncertainty analysis designed to identify parameters for which additional empirical data are most needed. Our analysis revealed important variation in output variables characterizing rabies dynamics, namely rabies persistence, exposure level, spatiotemporal distribution, and prevalence. Among epidemiological parameters, rabies transmission rate was the most influential, followed by rabies mortality and location, and size of the initial infection. The most influential landscape parameters included habitat-specific carrying capacities, landscape heterogeneity, and the level of resistance to dispersal associated with topography. Movement variables, including juvenile dispersal, adult fine-scale movement distances, and home range size, as well as life history traits such as age of independence, birth seasonality, and age- and sex-specific mortality were other important drivers of rabies dynamics. We discuss results in the context of mongoose ecology and its influence on disease transmission dynamics. Finally, we suggest empirical approaches and study design specificities that would provide optimal contributing data addressing the knowledge gaps identified by our approach, and would increase our potential to use epidemiological models to guide mongoose rabies control and management in the Caribbean.

## 1. Introduction

Small Indian mongooses (*Urva auropunctata*) were introduced from Asia to 29 Caribbean islands during the 19th century, primarily to control rodent populations on sugar plantations [1,2]. This opportunistic carnivore rapidly became invasive in Caribbean ecosystems, where it caused substantial damage to native fauna [3]. Moreover, in Puerto Rico, Cuba, Grenada, and the Dominican Republic, mongooses are the primary reservoir for canine rabies [4,5,6]. The first rabies outbreak in the Western hemisphere attributed to the small Indian mongoose was reported in Puerto Rico in 1950 [7]. Mongooses are now responsible for >45% of reported rabies cases in Puerto Rico [8], and during 2005–2008, 97% (*n* = 151) of specimens submitted after biting tested positive for rabies [9]. This species therefore represents a significant and persistent public health threat.

Phylogenetic studies revealed that rabies virus from mongooses in Puerto Rico, Cuba, and Grenada are derived from independent introductions of canine rabies virus [5,10,11,12]. The contrast between the restricted distribution of rabies virus in small Indian mongooses and the wide geographic distribution of this reservoir species within the Caribbean has tentatively been attributed to the historical absence of the virus in local dog populations, and to low mongoose densities on some islands (e.g., Trinidad) that might have prevented initial viral spill-over or viral persistence within mongoose populations [13]. However, these hypotheses remain untested and, to date, no study has examined the ecological conditions required for mongoose rabies persistence in the Caribbean. This represents an important knowledge gap because developing effective control strategies for wildlife diseases relies on understanding the disease ecology and the transmission dynamics within reservoir species [14].

Epidemiological modeling of disease–host systems offers an opportunity to increase our understanding of disease systems [15], can simulate and optimize disease control strategies [16,17,18,19], and provides a basis to estimate and forecast spatiotemporal risks to public health [20]. As landscape heterogeneity and animal behavior can significantly affect disease dynamics in wildlife populations [21,22,23,24,25], individual-based, spatially explicit models are increasingly used in epidemiological studies. However, small changes in parametrization of such models can result in significant variation in model outcomes [19,26,27]. Therefore, uncertainty or lack of available data regarding important model parameters in a system can significantly impact the accuracy of model predictions.

The model-guided fieldwork (MGF) framework [28] provides guidelines for wildlife disease ecology research by promoting collaboration between biologists and modelers to ensure that empirical studies collect information on important model parameters and that models are data-driven and appropriate to the study system. The MGF framework prompts scientists to use model sensitivity analysis to inform the design of field studies, addressing aspects of the system that are poorly understood and focusing data collection effort on highly sensitive parameters. Although traditional sensitivity analysis explores the entire parameter space, local sensitivity analyses (hereafter referred to as uncertainty analyses [29]), in which parameter ranges are determined from previous knowledge (e.g., restricted to parameter sets and ranges for which available data are scarce or uncertain) can be useful [30,31].

Recent research on mongoose rabies in the Caribbean has focused on obtaining empirical estimates of small Indian mongoose ecology, such as population density estimates, serosurveys investigating rabies exposure, and home range size estimates [3,6,32,33,34]. In addition, some oral vaccines have been demonstrated to protect mongooses against rabies [35,36], and field studies showed that placebo baits were consumed by mongooses [37,38,39]. This work has helped better characterize the mongoose rabies system and propose potential control strategies. Nevertheless, several key ecological processes driving mongoose rabies dynamics remain to be described, and uncertainty bounds on some studied variables remain large. Specifically, better baseline data on habitat-specific mongoose movement, population dynamics, and spatial distribution is needed [33]. Applying the MGF framework to the mongoose rabies system by integrating available empirical data into epidemiological and statistical models could help identify the biological, ecological and epidemiological processes that are most important in driving rabies dynamics. The MGF framework promotes modeling as a tool in the iterative process of generating hypotheses, gathering empirical evidence, and refining hypotheses. The use of uncertainty analysis to guide ecological study design aimed at acquiring the necessary field data to improve models of mongoose rabies could thus increase our ability to use these models to simulate rabies control strategies, including vaccination and population reduction. Such simulations could in turn inform the design of optimized control studies (e.g., localized oral rabies vaccination, trap–vaccinate–release, or depopulation) that would provide validation data for the models.

In this study, we applied the MGF framework to develop an epidemiological model consolidating existing data on mongoose rabies ecology in order to guide future field studies and inform ongoing mongoose rabies management efforts. Specifically, we aimed to: (1) parametrize a spatially-explicit, individual-based model (IBM) using the data currently available on the mongoose rabies system, (2) use uncertainty analysis to identify parameters for which additional empirical data are needed (i.e., parameters to which the model is most sensitive), and (3) provide testable predictions about mongoose rabies ecology in the Caribbean.

## 2. Materials and Methods

### 2.1. The Ontario Rabies Model (ORM)

The Ontario Rabies Model (ORM) is a spatially-explicit IBM designed to investigate the effects of animal biology, infectious disease epidemiology, disease control strategies (e.g., vaccination, population reduction) and landscape characteristics on rabies dynamics. The model has been previously validated and used for modeling raccoon rabies in Canada [40,41]. The ORM structure and process are extensively described elsewhere [18,40,42], and some details are included in Appendix A.

The ORM structure and process are extensively described elsewhere [18,40,42]. Briefly, throughout their lifetime, simulated individuals are characterized by an identity number, sex, and parental identity. For each discrete model time step of one week, individuals are further characterized by their age, location, offspring identities, disease status (susceptible, incubating or infectious) and the identities of individuals with whom they were in contact. Individuals that recover from a rabies infection remain susceptible.

Demographic model processes are stochastic. Male and female mating pairs are formed at random from individuals concurrently located in the same 1km^2^ cell (area of the landscape characterized by a carrying capacity and a resistance to incoming and outgoing movements), and females have age-specific probabilities of producing a litter. Breeding can occur either once or twice a year, based on user-defined seasonal birth peaks. Offspring remain with their mother until the user-defined age of independence. Individuals are subjected to weekly age- and sex-specific natural mortality prior to resource-limiting mortality (i.e., when a cell exceeds its carrying capacity) and rabies-induced mortality. Animals are allowed to move from their cell once per year within a given age- and sex-specific range of weeks. Individual dispersal distances (number of cells) are drawn stochastically from age- and sex-specific distributions, while movement direction is determined randomly. Dispersal is completed within a one-week time step, with animals moving directly to their destination cell without any interaction with animals located in cells along their route. Incubating animals have a weekly chance of becoming infectious based on a user-defined incubation period distribution. On a weekly basis, infectious animals can transmit rabies to susceptible individuals with which they enter into contact, based on a user-defined proportion. Individuals are considered to be in contact with all other animals in their cell, as well as with a proportion of animals located in the six adjacent cells according to the extent of their home range overlap with neighboring cells. No disease-control strategy was implemented in this study.

Mongoose density and spatial distribution in their various disease states across the landscape result from the processes of reproduction, mortality, and dispersal operating on individuals. Similarly, spatial distribution of disease mortality and incidence emerge from the inter-individual disease transmission and infection processes operating on the susceptible animals. Model input includes a landscape, an initial georeferenced mongoose population, and a set of biological and epidemiological parameters. Information required to parametrize the model includes landscape attributes, species life history traits, and behavior (i.e., movement and intraspecific contacts) and rabies epidemiology processes (Table 1). There is substantial heterogeneity in the empirical data available to inform these different input parameters (Table 1).

#### 2.1.1. Landscape

**Figure 1:**
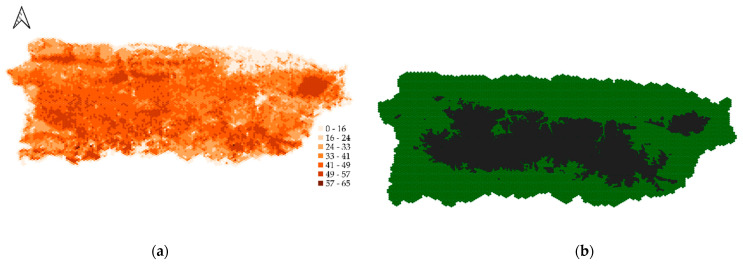
Experimental landscape of 203 × 134 one-km^2^ hexagonal cells representing the Puerto Rico main island. (**a**) Cell mongoose carrying capacities are the sum of the product of the proportion of the cell covered by each habitat type (derived from the USGS National Land Cover Database 2001 [53]) and its associated habitat-specific carrying capacity (Table 1). (**b**) Areas where elevation was ≥300 m (dark cells) were considered as potential barriers to mongoose dispersal.

The baseline landscape (Figure 1) was built by partitioning the main island of Puerto Rico (9104 km^2^) into 1 km^2^ hexagonal cells using the ORM_Landscape plugin in QGIS 2.18.20 [52]. This cell size represents the order of magnitude of maximal mongoose home range estimations [34]. We chose to model mongoose rabies on Puerto Rico because rabies is endemic in the mongoose population on this island, and most recent available empirical data on mongoose ecology is from this island (habitat-specific densities [6,33], rabies-virus serology [6], home ranges [34]). Five habitat types (semi-wooded, heavily wooded, open grassland, wetlands, and developed or barren land) were defined on the island based on resampling of the USGS National Land Cover Database 2001 [53] (Appendix A). The proportion of those five habitat types in each hexagonal cell was determined using the Zonal statistic plugin in QGIS. Individual cell carrying capacities for the baseline landscape were calculated as the sum of the product of the proportion of the cell covered by each habitat type and its associated habitat-specific carrying capacity (Table 1). Areas where elevation from sea level was ≥300 m (Figure 1b) were considered as potential barriers to mongoose dispersal [43] using a partial resistance to animal movement (Table 1).

#### 2.1.2. Initial Population

A mongoose population was generated on the baseline landscape initiated by a single mating pair and grown for 150 model years, which allowed population abundance on the landscape to stabilize. The resulting population was then used as the starting population for uniquely parametrized model simulations. This ensured that all simulations started with the same conditions before the unique parametrization of each model simulation changed model dynamics. At the beginning of each simulation trial, the model was run for five years prior to rabies introduction to allow population levels to adapt to the new cell carrying capacities associated with the trial.

**Figure 2:**
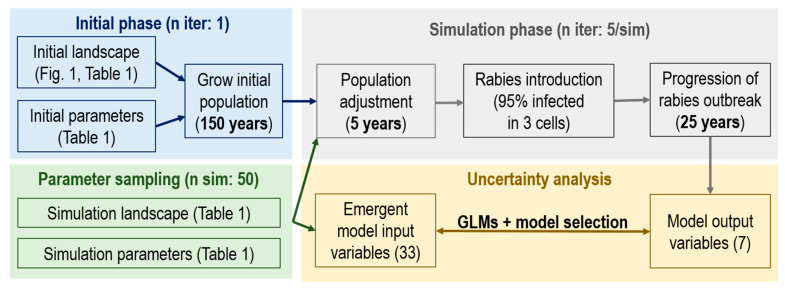
Conceptual workflow of Ontario Rabies Model (ORM) simulations and uncertainty analysis performed in this study.

On the first week of the fifth model year of the simulation, 95% of animals from three randomly selected, adjacent cells were infected with rabies. This represented 22.4 ± 0.7 animals/cell initially infected. The model was then run for a period of 25 model years to allow enough time for rabies to spread through the landscape and be regulated by disease dynamics associated with the trial characteristics (Figure 2).

#### 2.1.3. Input Parameters

Values for biological and epidemiological input parameters were determined based on published empirical data for the small Indian mongoose in the Caribbean. Parameters for which sufficient knowledge was available from the literature were defined as fixed parameters and assigned a constant value across all simulations (Table 1). Parameters for which significant uncertainty remains in current published studies were selected for uncertainty analysis. Ten model parameters were included in the uncertainty analysis: habitat-specific carrying capacities, effect of elevation on mongoose dispersal, age of independence and adulthood, age- and sex-specific annual mortality rates and dispersal distances, home range size, rabies transmission coefficient, rabies-induced mortality, and location and prevalence of initial rabies infection. These parameters represent the variables for which the effect of uncertainty from the literature on simulated rabies dynamics was assessed in this study.

#### 2.1.4. Parameter Value Sampling

For each variable parameter (*n* = 21), a probability distribution function (PDF) was determined based on data from reviewed literature, and a value was sampled from that PDF for each model simulation (Table 1 and Table 2). This procedure was repeated to generate a total of 500 unique parameter sets by Monte Carlo sampling. To account for stochasticity in model processes and outcomes, each parameter set was iterated five times using different starting seeds, for a total of 2500 model runs. Preliminary simulations indicated that five iterations were sufficient to capture the range of variation in model output attributable to stochasticity (*Var_stochasticity_*). Specifically,

(1) $$Var_{stochasticity}=\frac{\bar{CV_{iter}}}{CV_{sim}}$$

where *CV_iter_* and *CV_sim_* represent the coefficient of variation (CV) calculated among iterations of a same model parametrization and across all simulations, respectively. *Var_stochasticity_* was <0.15 for both duration of the rabies outbreak and time for the outbreak to cross half the length of the landscape.

#### 2.1.5. Output Variables

Outputs from the ORM were modelled as functions of simulation variable input parameters to assess landscape, biological, and epidemiological factors affecting the outcome of the epidemiological model. Six variables describing different aspects of the disease response to the model parametrization were extracted from the simulation output (Table 2).

### 2.2. Uncertainty Analysis

Thirty-four input variables describing the various aspects of the ORM parametrization were calculated (Table 3). Values directly sampled for model parametrization could not be used for this purpose because they would not have represented the model processes by themselves. For example, the average habitat carrying capacity (Kmean, Table 3) and carrying capacity coefficient of variation (Kcv) over the landscape emerged from the baseline K value used for the semi-wooded habitat, in combination with all coefficients used for the other habitat types during model parametrization, and represent attributes of model input parameters that are susceptible to directly influence rabies dynamics during simulation. The input variables integrated as fixed effects (Table 3) in the model selection detailed below were chosen because they described an emerging biological, behavioral, or epidemiological process that varied across simulations run in this study.

#### Model Selection

We fitted generalized additive models (GAM) to the response variables using distribution families relevant to the variable considered (Table 2; mgcv package [55]). Fixed effects (*n* = 33) considered for all six models represented ORM input parameters that were varied across simulations (Table 3). Running individual GAMs for each possible combination of fixed effects would have resulted in erroneous models because (1) some of the covariates were uninformative for the response variable considered, (2) some fixed effects were correlated with one another, and (3) this would have led to candidate model sets comprising >8.5 billion models. Therefore, we adopted a sequential explanatory modeling approach allowing for unsupported variables to be eliminated. 

The first step aimed at identifying covariates exerting some degree of influence on model output parameters using the null hypothesis testing approach [56]. Each fixed-effect variable was introduced as a linear term in univariate models, and only those having coefficient *p*-values <0.2 were retained in the fixed effect list for further steps [56]. This allowed the elimination of uninformative covariates, while conservatively keeping covariates displaying some degree of statistical support.

In a second step, we selected whether covariates were linearly or non-linearly related to the response variable. We fitted each retained fixed effect as a smooth term using thin plate regression splines as the smoothing basis and a maximal basis dimension of five in single variable GAMs. By definition, effective degrees of freedom (edf) equal one when the model penalizes a smooth term to a first-order linear relationship [55], fixed effects resulting in rounded smooth terms with edf ≤1 and >1 are considered to be linearly and non-linearly correlated to the response variable, respectively. Fixed effects linearly related to the response variable were thereafter introduced as linear terms rather than smooth terms in the GAMs, optimizing computational time and allowing for the estimation of their regression coefficient. Non-linear effects were reintroduced as smooth terms in further steps, with their basis dimension limited to their edf+1 from univariate models to avoid overfitting of smooth terms in multivariate models.

Because interdependence among explanatory covariates hampers model selection and regression parameter estimations [57,58,59], our third step consisted of detecting collinearity between pairs of covariates. Pairwise Pearson coefficients were computed for each pairwise combination of fixed-effect variables, and variable pairs with coefficients >0.6 were considered as significantly correlated. The aim of the present study was not to build models to be used for inference, but rather to identify the most influential variables shaping disease dynamics in an epidemiological model. Thus, we did not combine intercorrelated variables by principal component analysis (PCA), but opted to design our candidate model set such that only independent covariates were introduced together into multivariate models.

In a fifth step, we performed model selection using a sequential, information-theoretic approach [60]. The candidate set of GAMs included all possible combinations of uncorrelated fixed effects. No interaction term was included at this step to avoid exponentially increasing the size of the candidate model set by fitting interactions among variables not represented in top ranked models. To be considered as significantly improving a model, a given variable had to reduce the Akaike information criterion (AIC) by at least two points compared to the simpler model excluding the variable [60]. The model with the lowest AIC in which all covariates significantly improved the fit was retained as the top model of the candidate set [61]. This model-selection procedure was repeated independently for all six response variables investigated.

In a sixth step, fixed effect combinations ecologically or epidemiologically susceptible to interfering with one another were considered as potential interaction terms. These were restricted to the following: mongoose densities (Kmean and Kcv) and rabies transmission (Rab_spread_rate); movement distances, location of initial infection (Init_infect_x and Init_infect_y), and resistance to movement associated with elevation (Resist_elev); young-of-the-year (YOY) movement distances and age of independence; probability of being outside the home cell (Prob_OHC_M and Prob_OHC_F), Rab_spread_rate, and size of initial outbreak (N_init_infect); YOY natural mortality and the number of annual birth peaks; and sex- and age-specific natural mortalities and rabies-induced mortality. 

For each response variable, a second set of candidate models was built, which contained the top ranked model from step 5 to which all potential interaction terms among fixed effects represented in the model were added. This candidate model set also contained all combinations of nested models resulting from this “top ranked + all interactions” model. Interactions among linear terms were introduced as regular linear interactions (i.e., x1 × x2), while interactions between a smooth term and a linear term were introduced using the ‘by’ argument when defining the smooth in the GAM formulae. Lastly, interactions among smooth terms were represented using tensor product interactions (the *te* function from the mgcv package), where dimensions of the different bases (k argument in *te* function) were set to their respective smooth term k+1 value from the model without interactions [55]. As described above, the model with the lowest AIC in which all covariates and interaction terms significantly improved the fit was retained as the top model for each answer variable.

Data formatting and analysis was performed in the R environment [62]. Results are presented as means ± standard errors (SE), unless otherwise stated. Statistical significance was set at *p* < 0.05.

## 3. Results

**Figure 3:**
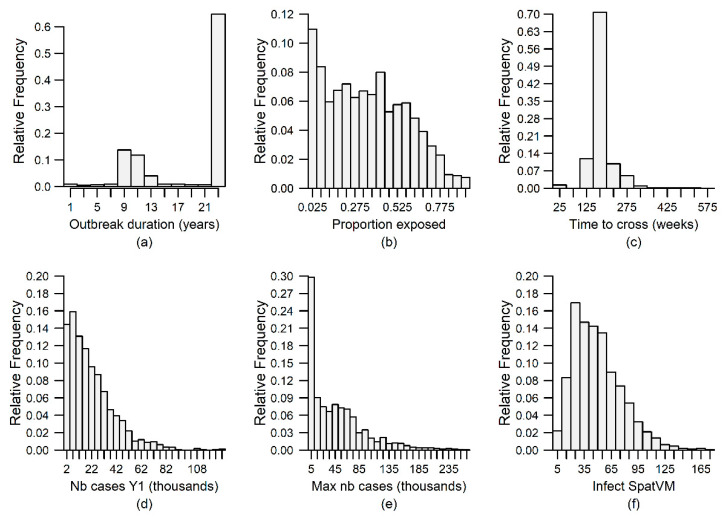
Distribution of six output variables extracted 24 years after the introduction of rabies on a landscape representing the Island of Puerto Rico populated by small Indian mongooses within the Ontario Rabies Model. Model parametrization was carried out using Monte Carlo sampling (*n* = 500) from the range of published data available on the mongoose rabies system. Simulations were iterated five times. Output variables are detailed in Table 3.

An epidemiological model adequately representing the mongoose rabies system is minimally expected to allow rabies to persist over time on the landscape, and result in a proportion of exposed animals no higher than what is observed in the field (i.e, rabies-virus-neutralizing antibodies (RVNA) seroprevalence <40%; [5,6,63]), combined with a low rabies infection prevalence. Those conditions were met in 906 (36.2%) of the simulations in this study. Among those 906 simulations, other output variables describing the rabies outbreak were highly variable (Figure 3) and most of this variation was attributable to model parametrization rather than to inter-iteration stochasticity (Table 2).

Duration of rabies outbreaks varied from zero to 24 years (mean = 19.4 ± 0.1, *n* = 2500). We defined “rabies persistence” as the occurrence of new rabies cases during every year of the 24-year-long simulation. Rabies persistence was observed in 1577 (63.1%) of the simulations. In simulations where rabies persisted, the proportion of populated cells hosting at least one rabid mongoose at the end of the simulation ranged from 0.01 to 36.82% (mean = 7.34 ± 0.09%, *n* = 1577), while the proportion of the total population that had been exposed to rabies in the course of their lifetime measured at the end of the simulation ranged from 0 to 94.9% (mean = 34.7 ± 6%, *n* = 1614). When the rabies outbreak expanded to at least 50% of the length of the study area, it took between 1 and 506 weeks to do so (mean = 175.3 ± 0.9 weeks, *n* = 2282). While the maximum weekly number of rabies cases (MaxInfect) ranged between 2 and 266 210 (mean = 45 552 ± 959, *n* = 2499), the number of cases one year after the initial infection (InfectY1) varied between 0 and 127 419 (mean = 22 213 ± 371, *n* = 2499). Finally, the spatial variance:mean ratio of infected animals (InfectSpatVM) ranged between 1.7 and 175.9 (mean = 49.6 ± 0.7). Since all six output variables displayed considerable variation, they were each used as response variables in GLMs with the aim of identifying which model input parameters contributed most to this variation.

The model selection procedure used in this study resulted in the selection of a single best model for each of the response variables (Table 4). All output variables examined were best explained by a combination of at least five response variables, including at least one movement, demographic, and epidemiological variable (Table 4). In addition, the best model describing the probability of rabies persisting in the population (Persistence), maximal weekly rabies incidence (MaxInfect), number of cases one year after the initial infection (InfectY1), the spatial variance:mean ratio of infected animals (InfectSpatVM) and the proportion of cells containing infected animals (%cell_infect) also included at least one landscape variable (Table 4).

**Figure 4:**
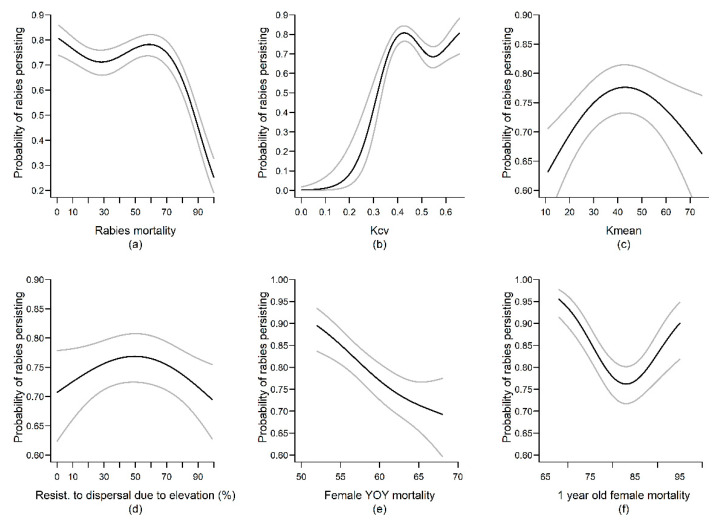
Effects of variation in model parameter values on the probability of a rabies outbreak persisting throughout the 24 years of simulation. Black and gray lines represent predicted values and 95% confidence intervals from the top-ranked model retained for the rabies persistence output variable (see Appendix B for details). Only smooth terms are shown.

The probability of rabies persisting in the population throughout the 25-year simulation was favored by intermediate habitat carrying capacities (Kmean) and intermediate resistance to movement associated with elevation, as well as high spatial heterogeneity in local (i.e, inter-cell) mongoose densities (Kcv; Figure 4). Moreover, rabies persistence was more probable when the initial infection occurred away from the island center on the North–South axis (Appendix C). Low female natural mortality, rabies-induced mortality, and rabies transmission rates facilitated rabies persistence (Figure 4, Appendix C). Rabies persistence also increased with distances moved by most sedentary mongooses on the landscape (ADL_movt_25pc; Appendix C). In contrast, large-scale movement by YOY (YOY_mvt_90pc) and delayed age of independence negatively affected rabies persistence (Appendix C).

**Figure 5:**
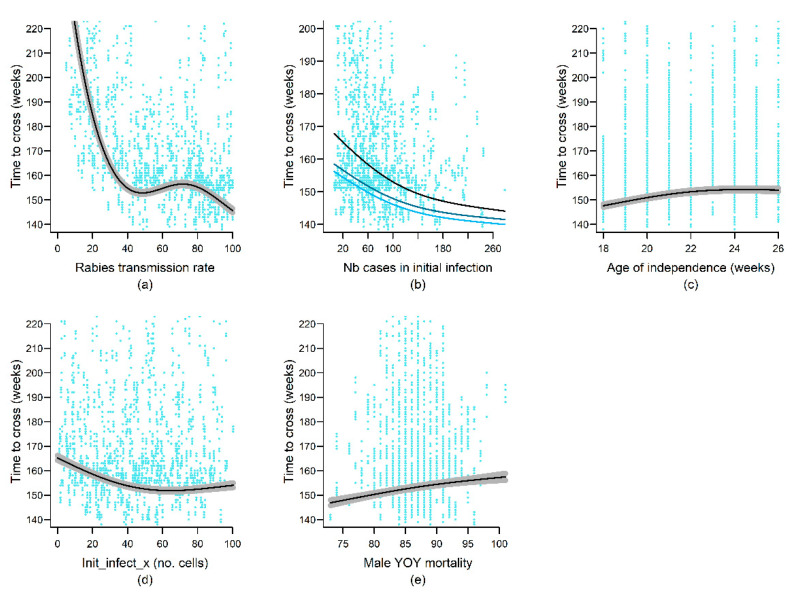
Effects of variation in model parameter values on the time the rabies outbreak took to travel through 50% of the length of the landscape (TimeToCross). Black and gray lines represent predicted values and 95% confidence intervals from the top-ranked model retained for the rabies persistence output variable (see Appendix B for details). Blue dots represent the ORM simulation results modeled by the generalized additive models (GAM). The blue gradient lines in (**b**) illustrate the interaction term, with lighter to darker blue representing increasing female probability of interacting with individuals outside their home cell (Prob_OHC_F). Only smooth terms are shown.

The time for the outbreak to cross half the length of the study area (TimeToCross) decreased with increasing distance moved by less mobile animals on the landscape (ADL_movt_25pc; Appendix C), suggesting that movement, even by most sedentary mongooses, facilitated transmission and played an important role in rabies spatial dynamics. Rapid progression of the rabies outbreak across the landscape was also favored by early ages of independence and low male yearling mortality (Figure 5), indicating that YOY and yearling mongooses were major contributors to rabies spread on the landscape. The rabies outbreak wave front travelled exponentially faster across the island as rabies transmission rates increased from zero to approximately 40% but further increases in rabies transmission rate had little impact (Figure 5). The severity of the initial infection (n_init_infect) was positively correlated with the speed of the rabies spatial spread, an effect that was influenced by local inter-specific contact rates among female mongooses (Prob_OHC_F). Finally, the rabies outbreak travelled faster across the island when the initial infection took place at a minimal distance from the island’s geographic center (>50 km; Figure 5), but not so close to the island coasts as to allow spatial spread in both directions on the East–West axis.

**Figure 6:**
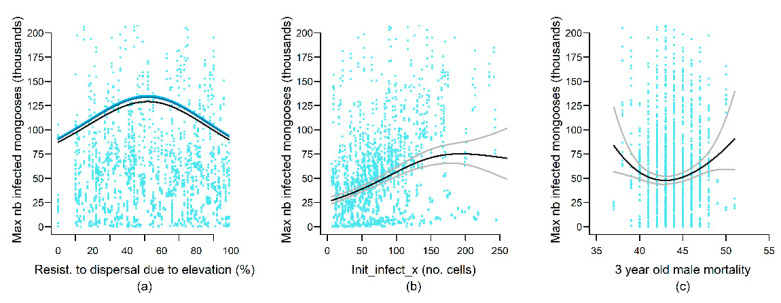
Effects of variation in model parameter values on the maximal number of infectious animals on the landscape at any given week following rabies introduction (MaxInfect). The blue gradient lines in (**a**) illustrates the interaction term, with lighter to darker blue representing increasing adult movement distances (Adult_mvt_75pc, see Appendix B for details). Black and gray lines represent predicted values and 95% confidence intervals from the top-ranked model retained for the rabies persistence output variable. Blue dots represent the ORM simulation results modeled by the GAM. Only smooth terms are shown.

**Figure 7:**
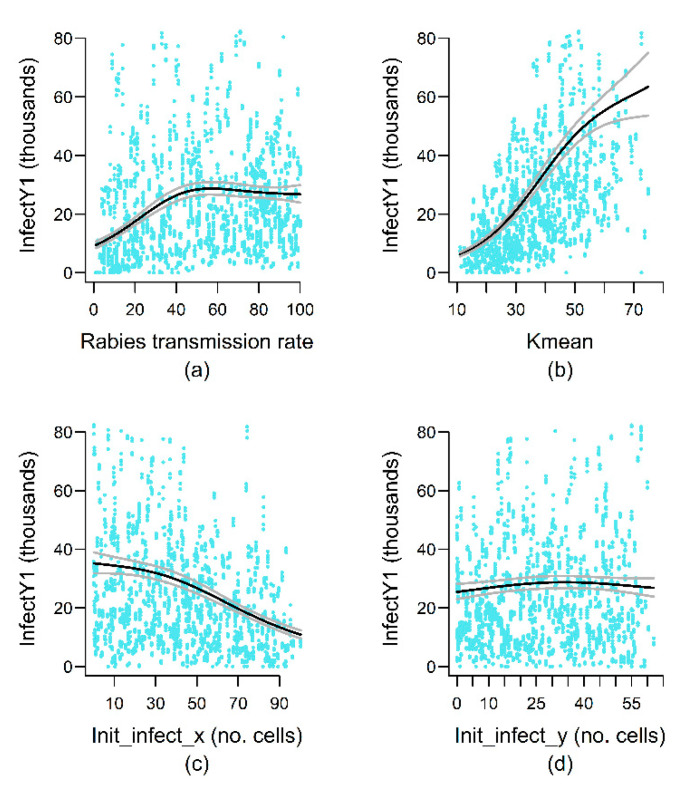
Effects of variation in model parameter values on the number of cases one year after the initial rabies introduction on the landscape (InfectY1). Black and gray lines represent predicted values and 95% confidence intervals from the top-ranked model retained for the rabies persistence output variable (see Appendix B for details). Blue dots represent the ORM simulation results modeled by the GAM. Only smooth terms are shown.

The maximal weekly rabies prevalence (MaxInfect) reached during the 25-year simulations was positively correlated with both adult and YOY dispersal distances, as well as the number of annual birth peaks, but negatively correlated with YOY and yearling mortality and age of independence (Appendix C). Increasing numbers of cases introduced as the initial infection (n_init_infect) lead to greater maximal weekly prevalence, but this effect plateaued at approximately 175 cases (Figure 6). The only landscape variable represented in the MaxInfect best model was the inter-cell resistance to movement associated with elevation (Resist_elev), with intermediate resistance values allowing greater infection prevalence. In contrast, rabies prevalence one year after rabies introduction on the landscape (InfectY1) was positively correlated with the average landscape carrying capacity (Kmean) and highly dependent on the location of the initial infection (Figure 7). InfectY1 also increased with greater distances traveled by less-mobile mongooses on the landscape (ADL_movt_25pc), low female YOY mortality, and delayed age of independence (Appendix C). Lastly, increasing rabies transmission rate from 0 to approximately 50% resulted in higher InfectY1, but this effect plateaued at greater transmission rates (Figure 7).

The InfectSpatVM is defined as the mean number of rabies cases per cell divided by inter-cell variance. High levels of InfectSpatVM therefore represent high rabies prevalence over the landscape, as well as low spatial heterogeneity. This variable was positively affected by the average cell carrying capacity across the landscape (Kmean), as well as high rabies transmission rates, delayed offspring independence, and high female YOY survival. InfectSpatVM was also greater in simulations where there was only one birth peak annually, compared to two birth peaks. Surprisingly, InfectSpatVM was positively correlated with the level of spatial heterogeneity in mongoose densities over the landscape (Kcv). This suggests that high heterogeneity in mongoose densities over the landscape increases average rabies prevalence to a point where this effect overrides the increased spatial heterogeneity in rabies cases per cell.

**Figure 8:**
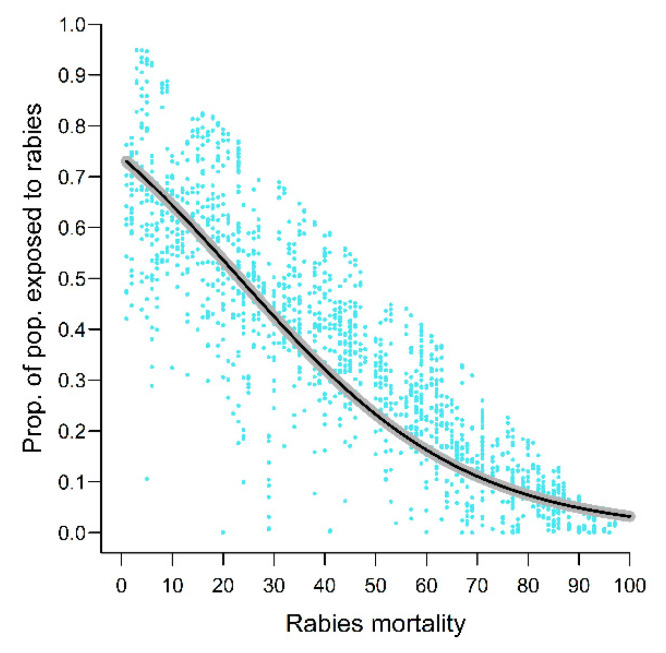
Effect of rabies mortality rate on the proportion of the mongoose population exposed to rabies 24 years after rabies introduction in the mongoose reservoir. Black and gray lines represent predicted values and 95% confidence intervals from the top-ranked model retained for the %Exposed output variable (see Appendix B for details).

The proportion of exposed animals (i.e., that were infected by rabies and recovered) on the landscape by the end of the 25-year simulation (%exposed) depended on a combination of animal movement variables, including inter-specific contact rates (Prob_OHC), as well as demographic and epidemiological variables (Appendix C). Notably, rabies-induced mortality alone explained 75.2% of the deviance in the %exposed models (Figure 8). Inclusion of additional covariates, especially terms interacting with rabies-induced mortality, resulted in a model with an explained deviance of 89.7%. No landscape variable was retained in the top ranked models for %exposed, indicating that disease characteristics and, to a lesser extent, animal behavior and life history outweigh any potential influence of spatial distribution of mongooses on individual probability of exposure to rabies.

## 4. Discussion

In this study, we parametrized a spatially-explicit individual-based model for the mongoose rabies system. Our epidemiological model is a valuable tool for rabies management, providing a platform to compare the potential outcomes of different infectious disease control methods including vaccination, population reduction and fertility control [42]. However, our ability to simulate rabies control strategies is currently limited by uncertainties related to model parametrization. We thus performed an uncertainty analysis designed to examine the implications of current knowledge gaps related to mongoose ecology, behavior, and rabies dynamics. Our analysis revealed important variation in six output variables considered, which was markedly greater among simulations than within iterations. This indicates that variation in model output is mostly attributable to the parameter ranges, rather than to stochasticity inherent to the model. Moreover, certain simulations led to unrealistic results (e.g., rabies not persisting), highlighting the need to refine model parametrization. Future empirical studies providing data on key drivers of mongoose rabies dynamics identified in this study would thus increase the value of epidemiological modeling as a tool supporting mongoose rabies control and management. To this effect, in Table 5 we provide specific recommendations regarding empirical approaches and study design specificities (variables to measure, sampling design) that would provide optimal contributing data to increase our current knowledge of the mongoose rabies system by addressing the most influential parameters on rabies dynamics identified by the uncertainty analysis described in the study.

### 4.1. Landscape Variables

Landscape variables were retained in regression analyses modeling rabies persistence, total number of cases one year after the initial infection (InfectY1), and InfectSpatVM (Table 4). Among landscape variables, the average habitat carrying capacity was the most frequently retained, followed by inter-habitat density variation and resistance to movement associated with elevation. Interestingly, mongoose carrying capacity at the location of the initial rabies outbreak was not retained in any models. This suggests that the distribution of mongooses at the landscape scale has a greater influence on rabies dynamics than fine-scale habitat characteristics of the location of rabies introduction. Thus, reliable habitat-specific mongoose densities combined with island land cover data from rabies-free Caribbean islands may be sufficient to investigate probability of rabies persistence and spatial dynamics in the mongoose reservoir if rabies was introduced. In our study, a broad range of island-wide mongoose averaged densities (0.13–0.74 animals/hectare) enabled rabies persistence. This is consistent with the theory that rabies persistence in Puerto Rico is possible across the range of mongoose densities previously reported [33].

Cell carrying capacities used in this study were derived directly from habitat-specific mongoose density estimates from field studies in the Caribbean, ranging from 0.19 to 9.0 mongooses/hectare [32,33,48,64,65,66]. This difference in reported mongoose densities is attributable to (1) differences among Caribbean islands associated with island biogeography, (2) habitat-specific differences in resource availability, and (3) differences in experimental design and analysis methods among published studies. Standardized density estimation methods that account for differences across the various habitat types characterizing the Caribbean landscape would eliminate this third source of variation in mongoose density, thus refining island- and habitat-specific mongoose density estimates and increasing our modeling capacity for the mongoose rabies system. 

Resistance to movement associated with elevation affected both probability of rabies persistence and the maximal weekly prevalence during the outbreak. To our knowledge, no study has directly investigated the importance of elevation as a barrier to mongoose movement. One study in Southeast Asia reported that although some small Indian mongooses were observed at elevations up to 1200 m, most remained below 300 m [43]. However, small Indian mongooses in Southeast Asia are sympatric with the crab-eating and short-tailed mongooses, and distribution and niche patterns could partly result from inter-specific competition [43]. As small Indian mongooses are the only small carnivore species occurring in the Caribbean, they might not be under the same ecological pressures and could potentially exploit suboptimal habitats such as elevations >300 m more commonly.

Although some Caribbean islands are relatively flat, others are characterized by rugged terrain and towering volcanic mountain ranges. In Puerto Rico, the highest peak rises to 1338 m and is part of La Cordillera Central, which occupies >30% of the main island. Mongooses do occur in mountainous rainforests in the Caribbean, at densities similar or lower to those observed in costal habitats [33,66,67,68]. Despite the widespread distribution of the species, it is conceivable that the landscape on some Caribbean islands could shape mongoose dispersal movement and home ranges. Accordingly, decades after introduction of rabies into the wildlife reservoir in Puerto Rico, geographical segregation of virus strains persists, suggesting a restrictive effect of La Cordillera on virus spatial distribution [11].

Mongoose rabies was reported in Dominican Republic (DR) but not in neighboring Haiti [69], although these countries share a land boundary. Whether this is representative of the epidemiological situation on Hispaniola Island, or a result of under-reporting of rabies cases [69] and of the challenges experienced during trapping efforts targeting the Haitian mongoose population [70] is not known. Mongoose densities in Haiti are likely lower than those in DR due to extensive deforestation, and potentially insufficient to support rabies persistence. Alternatively, five of the six peaks exceeding 2000 m in the Caribbean are located on Hispaniola, and the central mountain range spans from northwestern Haiti to the south coast of DR. Studies examining the influence of elevation on mongoose movement and dispersal could provide valuable insights into the role of island topography on mongoose rabies dynamics in the Caribbean, and allow the use of epidemiological models to investigate the probability of rabies outbreaks occurring in a specific region spreading across the island landscape and associated topographic barriers.

### 4.2. Movement Variables

It is not surprising that YOY movement had greater influence than adult movement on rabies dynamics in our study, since YOY were allowed to disperse up to ten times further than adults (Table 1 and Table 4). We opted for this differential parametrization among age groups because, although natal dispersal by YOY small Indian mongoose has not been described [49], it cannot be excluded given the lack of movement data for YOY mongoose [50,51]. The high heterogeneity in social systems among mongoose species advises caution when inferring life-history and behavioral traits from related species [71]. Accordingly, dispersal is mainly natal in dwarf mongooses [72], while banded mongooses mostly disperse as young adults aged 1–3 years [73]. Fieldwork studies designed to characterize natal dispersal in small Indian mongooses would allow refining the parametrization of juvenile dispersal, and correspondingly enhance our capacity to reliably model rabies dynamics in this species.

Extreme (i.e., 90th percentile and/or maximum) YOY movements were important predictors for rabies persistence, %Exposed, TimeToCross and MaxInfect. This suggests that long-distance juvenile dispersal events, even if uncommon, can have a major impact on rabies dynamics. In contrast, movement distances of most sedentary adult animals (25th distance percentile) were retained in more models than distances moved by highly mobile adults (e.g., 90th percentile and maximum; Table 4). These results suggest age-class specific roles in rabies dynamics, with YOY propagating the virus over long distances and generating new outbreaks on the landscape during the epidemic phase, and adults driving the local transmission and progression of the disease wave front. Interestingly, this effect is similar to the vampire bat rabies system, where sex-biased dispersal results in a disproportionate role of males in spatial spread of rabies between isolated populations [74].

Probability of being outside home cell (Prob_OHC) was positively correlated with the proportion of the mongoose population exposed to the virus, and interacted with the initial number of infected animals to determine spatial spread. This variable is derived from the ratio between mongoose home range sizes and the size of cells forming the virtual landscape. Mongoose home range estimates in the Caribbean vary greatly among studies, from 1 ha to >50 ha [34,64,75,76]. Home ranges are 1.2 to 9.8 larger for males than females [34]. Among these studies, methodologies used for home range estimation, study duration, and habitat types varied considerably, making comparisons difficult. Standardization of estimation methods and study site selection accounting for the different Caribbean habitat types would therefore increase the accuracy and precision of mongoose rabies dynamics simulations.

### 4.3. Demographic Variables

Among demographic variables, the age of juvenile independence was retained in top ranked models for all six output variables, indicating that it influenced spatiotemporal distribution of rabies cases throughout the outbreak. Generally, early offspring independence resulted in higher probabilities of rabies persistence, faster progression of the outbreak over the landscape, and higher prevalence in the population. In our epidemiological model, females that were infected with rabies automatically infected their dependent offspring. When maternal care duration is extended, young that were infected from maternal exposure have higher chances of going through the incubation and infectious periods during their dependent phase. In contrast, when maternal care is short, a greater proportion of juveniles are released into the general population during the incubation or infectious phases. As reproduction is synchronous across the landscape, the end of the parental care period can coincide with a massive release of new susceptible and infectious individuals into the population. Some of these animals are highly mobile due to natal dispersal, allowing rapid spatial spread of the virus. Likewise, a greater number of annual birth peaks resulted in increased exposure to the virus, higher maximal prevalence, and reduced spatial heterogeneity in prevalence. This is not surprising, as the number of annual birth peaks determines the frequency at which a cohort of juvenile mongooses is released into the population. Similarly, it was demonstrated that annual birth synchrony, via its effect on intra-annual population size variation, can drive infectious disease dynamics, especially in species with high demographic turnover [77]. 

In captivity, young mongooses follow their mothers until they are four to six months of age [44]. To our knowledge, no study of free-ranging mongooses has estimated age of independence. Given the influence of age of independence on every aspect of rabies dynamics uncovered by our results, we suggest that further research on ontogeny of parental care and social interactions in this species would be valuable. Similarly, the possibility for female mongooses to breed twice a year in our model is based on the observation that a captive individual on St. Croix produced two litters within a four months interval, and on population reproduction data indicating that two to three birth peaks occur annually [44]. It remains to be confirmed what proportion of females breed more than once a year in the wild. Moreover, information on life-history traits, such as whether survival of her litter influences female breeding activity and maternal care allocation the following months has not been reported and would be valuable as it is likely to have impacts on rabies dynamics within the population.

Juvenile and first year mortality rates were also important explanatory variables, with female mortality retained more often in models than male mortality. The mongoose mating system is promiscuous and males provide no parental care [44,71]. Given the importance of recruitment for rabies dynamics detailed above, it is not surprising that female mortality had greater influence than male mortality on model output. To our knowledge, natural mortality rates among free-ranging mongooses have not been documented. Baseline mortality values used to parametrize the model were inferred from an age structure histogram from a mongoose study in St. Croix [44]. As mortality is likely to vary with resource availability and intra- and inter-specific interactions, studies designed to monitor mongoose survival in free-ranging populations from different Caribbean habitats could improve our understanding of factors driving mongoose demographic dynamics.

### 4.4. Epidemiological Variables

At least one epidemiological variable was retained in all final regression models, and the rabies transmission rate (Rabies_prob_spread) was the variable most frequently retained. In our model, an infected animal interacts with all other animals within its current cell, as well as with a proportion of individuals in the six adjacent cells [42]. Of those interactions, a proportion (defined as Rabies_prob_spread) result in rabies transmission. Therefore, the rabies transmission rate in our model represents two processes: (1) the intra-specific contact rate among mongooses with overlapping home ranges, and (2) the probability of an infected individual transmitting rabies given a significant contact. Although it was reported that mongoose home ranges from both sexes extensively overlap [34,51], no study has quantified intra-specific contacts rates or rabies transmission rates upon contacts in a free-ranging population. Such information, although difficult to obtain, would greatly improve our ability to model mongoose rabies dynamics. 

The prevalence and location of the initial rabies outbreak were also determinants of several disease dynamics variables (Table 4). It may seem counterintuitive that the rabies outbreak progressed more rapidly over the landscape and had higher probabilities of persistence when the initial infection was located farther from the center of the island. However, this might be attributable to the restrictive effect of the central Cordillera range on rabies transmission, therefore providing additional evidence for the role of elevation on mongoose rabies dynamics. In Puerto Rico, rabies was present in dogs and farm animals as early as the 1930s, and abruptly emerged in mongooses in 1950 at different scattered locations across the island [7]. Molecular evidence indicates that the virus was independently introduced twice in the mongoose reservoir [11]. However, it is not possible to identify the location where the initial mongoose infections took place and how many individuals were initially affected. In the Caribbean, tourism and importation of companion animals are likely mechanisms of canine rabies introduction [69]. Our study suggests that the modeling approach developed here could be applied to assess the risk that a localized introduction event, occurring at various high-risk areas (e.g., ports, touristic cities, airports), would result in rabies establishment on different Caribbean islands.

Rabies-induced mortality was an important determinant of disease persistence and of the proportion of animals exposed on the landscape. Mongoose rabies has been endemic in Puerto Rico, Dominican Republic, Cuba and Grenada since at least the 1950s [69]. In this study, a broad set of mongoose demographic and life-history traits resulted in high probabilities of rabies persistence on the landscape. However, rabies did not persist in any simulation where rabies-induced mortality was >97%. This indicates that rabies persistence among mongooses in the Caribbean depends on a certain level of nonlethal exposures, and is otherwise robust to a range of pathological and ecological assumptions. This conclusion is similar to that reported by Blackwood et al. [78], where rabies persistence in vampire bats was primarily determined by frequent (around 90% of the population) immunizing but non-lethal rabies exposures as well as immigration of infectious individuals from neighboring populations. In contrast, lower frequencies of non-lethal exposure in mongooses may facilitate rabies persistence in this rapidly reproducing species.

Although the probability of developing a lethal infection upon exposure is considered much higher in carnivores than in bats [79], non-lethal exposure in mongoose rabies might play a crucial role in the disease dynamics. This suggestion is supported by empirical findings of relatively high prevalence of positive rabies RVNA in apparently healthy, unvaccinated mongooses in the Caribbean. RVNA seroprevalence ranging between 19.3–39.3% were reported in Puerto Rico and Grenada [5,6,63], while prevalence of mongooses positive for rabies virus was 1.7% [5] and 1.3% [80] in Grenada. In Puerto Rico, Berentsen et al. [6] detected no active shedding of rabies virus in mongoose saliva (*n* = 147), despite a RVNA prevalence of 39.3%. Levels of circulating RVNA from wild-caught mongooses were reported to decrease over time, but persisted >35 months in some individuals [80]. However, in the absence of appropriate cut-offs and long-term, species-specific studies examining how long RVNA last in wildlife populations, the proportion of animals seropositive for RVNA is unlikely to provide an accurate estimate of the prevalence of nonlethal rabies exposure [81]. 

### 4.5. Study Limitations

Individual-based models are faced with a trade-off between computational complexity, and ecological accuracy. Because mongooses are similar to raccoons in terms of movement behavior and social system, parametrization of the ORM for the small Indian mongoose was fairly straightforward. The main limitation was the high degree of uncertainty associated with several parameters, as represented by the parameter ranges and distributions used in the uncertainty analysis detailed in this study.

Nevertheless, some assumptions were made in the ORM that might affect model output. Firstly, the model assumes that animal behaviors are the same year-round, and that infected and infectious individuals behave like non-infected animals. This is unlikely to be realistic, as rabid mongooses have been described to undertake unprovoked attacks towards humans, to display altered circadian activity patterns, and to behave aberrantly [7,44]. Such virus-induced aggression and behavior is likely to increase intra- and interspecific contact rates in infectious individuals, and hence rabies transmission. Moreover, in banded mongooses, dispersing individuals are more frequently involved in aggressive interactions than individuals in established groups [73]. Quantification of the influence of individual attributes such as age, movement, and epidemiological status on mongoose behavior and activity would allow the incorporation of state-specific animal behavior in the ORM. Secondly, animal movement direction was random over the landscape. However, mongooses are known to congregate to forage on anthropogenic food sources and animal carcasses [51] with important potential consequences for rabies transmission. Further quantification of mongoose fine-scale aggregation around locally abundant resources could guide the integration of specific movements towards attraction points on the landscape, and thus improve capacity to accurately model the mongoose rabies system.

## 5. Conclusions

This study suggests that additional knowledge related to mongoose densities, movement, survival, and rabies epidemiology would considerably improve the parametrization of epidemiological models of mongoose rabies in the Caribbean. The resilience of rabies persistence to a broad combination of landscape, demographic, and life-history traits suggests that elimination from the mongoose reservoir may be particularly challenging, reinforcing the need for properly parametrized, reliable epidemiological models. We suggest that using results from our study to design future ecological fieldwork would provide important data to increase our capability to model mongoose rabies dynamics, and accordingly improve our potential to use epidemiological models to simulate mongoose rabies control strategies and guide management programs across the Caribbean. Knowledge gaps related to the most influential parameters identified in our study provide useful targets for empirical studies (Table 5) advancing the field of mongoose rabies research.

## Figures and Tables

**Table 1:** Parameterization of the Ontario Rabies Model (ORM): fixed values (light cells) and range of parameter variation (shaded cells) included in the uncertainty analysis. Parameter values in uncertainty analysis simulations were selected within their range using a uniform distribution unless otherwise stated.

Parameter	Initial Value (Population Growth)	Value	Reference
Biological parameters			
Habitat-specific carrying capacities (animals/km^2^)	Semi-wooded: 80 Heavily wooded: 50 Grassland: 30 Wetlands: 10 Developed: 4	Baseline: semi-wooded range: 33–124 animals/km^2^. Coefficient ranges: -Wooded: 0.1–1.52 -Heavily wooded: 0.43–1.087 -Grassland: 0.1–0.43 -Wetlands: 10 -Developed: 4	Mean densities presented in [33], relative difference between habitats from [32]. No available data to inform coefficient values for developed areas and wetlands.
Elevation-related resistance to movement	Resistance to movement when cell elevation ≥300 m: 0%	Resistance to movement when cell elevation ≥300 m range: 0–99%.	Small Indian mongooses mostly stay at elevations <300 m [43]
Age of independence (mongoose becomes juvenile, no longer dependent on maternal care)	22 weeks	Range: 17.5–26.3 weeks	[44,45]
Age of adulthood (mongoose no longer juvenile, i.e., sexually mature)	22 weeks	Range: 1.1–42.6 weeks after age of independence	[44]
Age- and sex-specific average annual mortality (%)	0 year: 35.9 (M); 60.1 (F) 1 year: 86.4 (M); 80.9 (F) 2 years: 58.8 (M); 84.6 (F) 3 years: 43.9 (M); NA (F)	For animals aged <4 years, selection from normal distribution where mean= initial age- and sex-specific mortality rate and SD = 0.05 × mortality rate For animals aged ≥4 years, selection from uniform distribution, range: 40-95	Derived from population age distributions in [44]
Gestation period	7 weeks	7 weeks	[46]
Distribution of birthing date	mean = week 27 (first week of July), SD = 2 weeks	1st birth peak: mean = week 27 (first week of July), SD = 2 weeks 2nd birth peak (if present): week 1 (first week of January), SD = 2 weeks	Derived from birth distribution in [44]
Number of annual birth peaks	1	2 if maternal care period allows it, 1 otherwise	[44]
Prevent mating of siblings?	No	No	No published data suggesting such a mechanism
Female juvenile birth probability	50	50	[47]
Female adult birth probability	100	100	[47]
Average litter size +/− variance	4 +/− 4	4 +/− 4	[44,48]
Litter M:F sex ratio	1:1	1:1	[44]
Weeks when dispersal is permitted	Week 19 (2nd week of May)	Week 19 (2nd week of May)	[49]
Age- and sex-specific dispersal distance	For both sexes and all ages: 50% probability of moving 1 cell; 50% probability of moving 2 cells	Gamma distribution with: Juveniles: Mu range: 1–10; scale range: 0.1–10 Adult females: Mu = 1, scale range: 0.1–10 Adult males: Female movement distribution × 1.46	Derived from [50,51]
Probability of interaction with animals from neighboring cells	For both sexes: 22% (constant throughout the year)	Probability obtained based on home range sizes ranging from 0.01 km^2^–0.5 km^2^ for females. Multiplying factor for male home range: 2.05–2.48.	[51]
Epidemiological parameters			
Distribution of rabies incubation period	NA	1 week: 25% 2 weeks: 25% 3 weeks: 25% 4 weeks: 25%	Computed from empirical data presented in [7]
Duration of rabies infection period	NA	2 weeks	Computed from empirical data presented in [7]
Rabies transmission coefficient (probability of transmission given a contact between infectious and susceptible individuals)	NA	Range: 1–100%	No published data
Rabies-induced mortality	NA	Range: 1–100%	No published data
Initial infection location	NA	3 adjacent cells randomly selected on landscape	No published data; rabies introduction in islands considered isolated events
Initial infection prevalence	NA	Range: 50–100%	No published data; rabies introduction in islands considered isolated events
Time of initial infection	NA	1st week of 5th year of simulation	Rabies introduced following time required for population size to adjust to landscape carrying capacities

**Table 2:** Model output parameters used as response variables in the uncertainty analysis.

Variables	Type of Variable	Description	Distribution	No. var ^1^(P)	No. Models	n	*Var_stochasticity_*
Persistence	Logical (binary)	Whether rabies persisted (1) or not (0) from initial infection through the end of the 25-years simulation.	Multinomial logistic regression (package nnet [54])	20	131,072	2500	0.1173 ^2^
%exposed	Proportion	Proportion of the total population that was exposed to rabies and recovered from the infection at the end of the 25-years simulation (overall indicator of outbreak severity)	Binomial	22	1,262,144	1614	0.3099
TimeToCross	Latency time	Time (weeks) since initial infection for the disease to extend to half the length of the study area (measures the severity of the initial outbreak)	Gamma	23	1,408,579	2282	0.0842
MaxInfect	Positive integer (count)	Maximal number of rabies cases that occurred during any week of the simulation (overall indicator of outbreak severity)	Negative binomial	21	131,072	2499	0.0993
InfectY1	Positive integer (count)	Total number of rabies cases one year after the initial infection.	Negative binomial	22	262,144	2499	0.0998
InfectSpatVM	Positive numeric	Mean: variance ratio for the number of infected animals per cell (spatial measure of diseased population dynamics)	Gamma	25	262,144	1573	0.1388

^1^ Number of significant variables retained from the *p*-value > 0.2 criteria; ^2^ Computed for outbreak duration in years as variation coefficients could not be calculated for the logical persistence variable.

**Table 3:** Variables considered as potential fixed effects in the model selection procedure aimed at identifying the ORM input parameters that are most influential for model output.

Variable	Description
Landscape-related variables	
Kmean	Average cell carrying capacity on the landscape
Kcv	Coefficient of variation of cell carrying capacities over the landscape
Resist_elev	Resistance to incoming and outgoing movement (%) among cells where elevation ≥300 m above sea level, representing the impermeability index of the barrier to mongoose dispersal associated with landscape elevation
K_init_infect	Sum of the carrying capacities from the three cells where the initial rabies infection occurred
Animal movement variables	
YOY_Max_Mvt	Young of the year maximum annual movement distance allowed
Adult_Max_Mvt	Adult maximum annual movement distance allowed
YOY_mvt_0pc YOY_mvt_25pc YOY_mvt_50pc YOY_mvt_75pc YOY_mvt_90pc	Young of the year 0th, 25th, 50th, 75th, and 90th percentiles of annual movement distance allowed
Adult_mvt_25pc Adult_mvt_50pc Adult_mvt_75pc Adult_mvt_90pc	Adult 0th, 25th, 50th, 75th, and 90th percentiles of annual movement distance allowed
Prob_OHC_M Prob_OHC_F	Sex-specific weekly probability of being outside home cell (i.e., interacting with individuals from a neighboring cell)
Demographic variables	
Nb_birth_peaks	Number of annual birth peaks
Age_ind	Age (weeks) at which young of the year become independent from their mother and undergo demographic processes independently
YOYM_mortality 1yrM_mortality 2yrM_mortality 3yrM_mortality 4yrM_mortality YOYF_mortality 1yrF_mortality 2yrF_mortality 3yrF_mortality 4yrF_mortality	Sex- and age-specific annual mortality rates for animals aged <1, 1, 2, 3, and 4 years old
Epidemiological variables	
Rab_spread_rate	Probability of an individual transmitting rabies when interacting with a conspecific
Rab_mortality	Rabies-induced mortality rate
Init_infect_x Init_infect_y	Vertical (y) and horizontal (x) distance (no. of cells) from the landscape center point where the initial rabies infection occurred
N_init_infect	No. of animals infected by the initial rabies infection

**Table 4:** Response (model output) and predictor (model input) variables used in the uncertainty analysis. Plus (+), minus (−), and nl indicates that the predictor was represented in the top-ranked model retained from the model selection procedure, and positively, negatively, or non-linearly correlated to the response variable, respectively. The asterisk represent an interaction between variables.

	Response Variables	Persistence	%Exposed	TimeTo Cross	InfectY1	MaxInfect	Infect SpatVM
Landscape variables	Kmean	nl			nl		nl
	Kcv	nl					+
	K_init_infect						
	Resistance_in/out	nl				nl	
Animal movement variables	YOY_mvt_0pc						
	YOY_mvt_25pc						
	YOY_mvt_50pc						
	YOY_mvt_75pc						
	YOY_mvt_90pc	−	+			+	
	YOY_Max_mvt		nl	+			
	Adult_mvt_25pc	+			+	+	
	Adult_mvt_50pc						
	Adult_mvt_75pc		+			nl	
	Adult_mvt_90pc		+				
	Adult_Max_Mvt						
	Prob_OHC_M		−				
	Prob_OHC_F		+	nl			
Demographic variables	Nb_birth_peaks		+			+	−
	Age_ind	−	nl	nl	+	-	nl
	YOYM_mortality		−				−
	1yrM_mortality		nl	nl			
	2yrM_mortality						
	3yrM_mortality		+			nl	
	4yrM_mortality		nl				
	YOYF_mortality	nl	nl		−	−	
	1yrF_mortality	nl	nl			−	
	2yrF_mortality						
	3yrF_mortality		+				
	4yrF_mortality	−					
Epidemiological variables	Rab_spread_rate	−	nl	nl	nl		nl
	Rab_mortality	nl	nl				
	Init_infect_y	+			nl		
	Init_infect_x			nl	nl		
	N_init_infect		+	nl		nl	
Interaction terms		none	YOY_mvt_90pc* Age_ind 4yrM_mortality* YOYF_mortality* 1yrF_mortality * Rab_mortality	Prob_OHC_F* N_init_infect	none	Adult_mvt_75pc* Resistance_in/out YOY_mvt_90pc* Age_ind	Kmean* Rab_spread_rate

**Table 5:** Model-guided fieldwork framework applied to the mongoose rabies system in the Caribbean: suggested empirical approaches and study design specificities (variables to measure, sampling design) that would provide optimal contributing data to increase our current knowledge of the mongoose rabies system by addressing the most influential parameters on rabies dynamics identified by the uncertainty analysis described in this study.

	Landscape Variables		Movement Variables			Demographic Variables			Epidemiological Variables
	Habitat-Specific Densities (Kmean, Kcv)	Resistance to Dispersal Associated with Elevation	YOY Dispersal	Adult Fine-scale Movement	Home Range Size	Age of Independence	Nb Annual Birth Peaks	Age- and Sex-Specific Mortality	Rabies Transmission Rate
Capture/Mark/ Recapture (CMR)	standardized method sampling in different habitats	sampling across a topographic gradient			standardized method sampling in different habitats	baited automatic cameras monitoring marked females (presence of pups)	female reproductive status upon capture (nursing, pregnant) baited automatic cameras monitoring marked females (presence of pups)	age estimation (age distribution curves) survival analysis from CMR data	
Telemetry		equipment of animals in mountainous areas	equipment of YOY	fine-scale location (e.g., GPS data, automated passive integrated transponder (PIT) tag scanning)	sampling in different habitats	equipment of pregnant females automated PIT tag scanning			proximity function
Genetics		genotype comparisons of populations across mountainous landscapes							

## Appendix A

**Table A1:** Habitat type definitions for the simulation landscape based on resampling of the USGS National Land Cover Database 2001 [53]. Pixel codes used in resampling were assigned either one of the five Caribbean terrestrial habitat types considered in this study.

Habitat Type	Pixel Codes Used in Resampling	Proportion of Puerto Rico Main Island (%)
Semi-wooden	52. Shrub/scrub	2.21
Heavily wooden	42. Evergreen forest	37.80
Open grass	21. Developed, open space 22. Developed, low intensity 71. Grassland/herbaceous 81. Pasture/hay 82. Cultivated crops	36.4
Wetlands	90. Woody wetlands 95. Emergent herbaceous wetlands	2.32
Developed or barren land	23. Developed, medium intensity 24. Developed, high intensity 31. Barren land (rock/sand/clay) 11. Open water	21.24

## Appendix B

**Table A2:** Top Akaike information criterion (AIC) ranked models satisfying the ΔAIC <2 criterion predicting each of the seven output parameters examined for the uncertainty analysis. Model output (response) and input (predictor) variables are detailed in Table 3 and Table 4, respectively. The asterisk represent an interaction between variables.

Response Variable	Predictors	AIC
Persistence	s(Kmean) + s(Kcv) + YOY_mvt_90pc + Adult_mvt_25pc + Age_ind + s(YOYF_mortality) + s(1yrF_mortality) +s(4yrF_mortality) + s(Rab_mortality) + Rab_spread + Init_infect_y + s(Resistance_in/out)	2946.73
%exposed	YOY_Max_mvt + te(YOY_mvt_90pc, Age_ind) + Adult_mvt_75pc + Adult_mvt_90pc + Prob_OHR_M + Prob_OHR_F + Nb_birth_peaks + YOYM_mortality + s(1yrM_mortality) + 3yrM_mortality + 3yrF_mortality + te(4yrM_mortality, YOYF_mortality, 1yrF_mortality, Rab_mortality) + Rab_spread_rate + N_init_infect	19542698
TimeToCross	YOY_Max_Mvt + s(Age_ind) + s(1yrM_mortality) + s(Rab_spread_rate) + s(N_init_infect, by = Prob_OHR_F) + s(Init_infect_x)	6250585815
MaxInfect	YOY_mvt_90pc + Adult_mvt_25pc + Age_ind + Nb_birth_peaks + s(3yrM_mortality) + YOYF_mortality + 1yrF_mortality + s(N_init_infect) + s(Resistance_in/out, by= Adult_mvt_75pc) + YOY_mvt_90pc* Age_ind	58277
InfectY1	s(Kmean) + s(Init_infect_y) + s(Init_infect_x) + Age_Ind + s(Rab_spread_rate) + YOYF_mortality + Adult_mvt_25pc	53425
InfectSpatVM	te(Kmean, Rab_spread_rate) + Kcv + s(Age_ind) + Nb_birth_peaks + YOYM_mortality	11968

## Appendix C

**Table A3:** Parameter estimates and statistics for the top-ranked model predicting the six output variables extracted from the Ontario Rabies Model (ORM) 25 simulated years after rabies introduction. The asterisk represent an interaction between variables.

Response Variable (Model Output Variables)	Fixed Effects (Model Input Variables)	β (Linear terms) or Edf (Smooth Terms)	β SE (Linear Terms)	Z Value (Linear Terms) or χ2 (Smooth Terms)	*p*-Value
Time to cross	s(Rab_spread_rate)	2.995	-	12143.8	<0.0001
	s(N_init_infect, by = Prob_OHR_F)	2.942	-	912.1	<0.0001
	s(Init_infect_x)	1.959	-	510.5	<0.0001
	s(Age_ind)	1.889	-	219.6	<0.0001
	s(M_motality_1yr)	1.452	-	139.2	<0.0001
	YOYF_Max_mvt	4.39e-5	0.0000	19.18	<0.0001
	Intercept	0.015	0.0005	28.39	<0.0001
Persistence	s(Rab_mortality)	2.970	-	165.240	<0.0001
	s(Kcv)	2.946	-	41.836	<0.0001
	s(F_mortality_1yr)	1.964	-	29.173	<0.0001
	s(Kmean)	1.919	-	16.715	0.0004
	s(YOYF_mortality)	1.656	-	23.476	<0.0001
	ADLF_mvt_25pc	1.015	0.3148	3.213	0.0013
	Age_ind	-0.061	0.0184	−3.329	0.0009
	YOYF_movt_90pc	-0.045	0.0104	−4.326	<0.0001
	Init_infect_x	0.010	0.0027	3.592	0.0003
	F_mortality_4yrs	-0.007	0.0029	−2.341	0.0192
	Rab_prop_spread	-0.005	0.0016	−2.897	0.0038
	Intercept	2.824	0.4918	5.741	<0.0001
%exposed	te(4yrM_mortality, YOYF_mortality, 1yrF_mortality, Rab_mortality)	190.982	-	94435503	<0.0001
	te(YOY_mvt_90pc, Age_ind)	23.998	-	994543	<0.0001
	s(Rab_prop_spread)	3.000	-	947939	<0.0001
	s(M_mortality_1yr)	1.998	-	138348	<0.0001
	YOYF_max_movt	0.025	0.0001	251.84	<0.0001
	ADLF_mvt_75pc	0.110	0.0005	201.12	<0.0001
	ADLF_mvt_90pc	0.023	0.0004	53.49	<0.0001
	Prob_OHR_M	−0.007	0.0001	−67.27	<0.0001
	Prob_OHR_F	0.037	0.0003	148.62	<0.0001
	Nb_birth_peaks	0.400	0.0010	397.74	<0.0001
	YOYM_mortality	−0.013	0.0009	−135.04	<0.0001
	3yrM_mortality	0.009	0.0000	95.49	<0.0001
	3yrF_mortality	0.001	0.0000	59.22	<0.0001
	N_init_infect	0.001	0.0000	154.22	<0.0001
	Intercept	−3.947	0.0195	−202.36	<0.0001
MaxInfect	s(3yrM_mortality)	2.605	-	20.16	<0.0001
	s(N_init_infect)	2.556	-	161.05	<0.0001
	s(Resistance_in/out, by= Adult_mvt_75pc)	2.971	-	37.70	<0.0001
	Nb_birth_peaks	0.319	0.0780	4.087	<0.0001
	ADL_mvt_25pc	0.314	0.1292	2.433	0.0150
	YOYF_movt_90pc	0.080	0.0435	1.844	0.0651
	YOYM_mortality	−0.036	0.0076	−4.650	<0.0001
	F_motality_1yr	−0.013	0.0055	−2.388	0.0169
	YOY_mvt_90pc* Age_ind	−0.004	0.0020	−2.141	0.0322
	Age_ind	–0.002	0.0243	–0.101	0.9195
	Intercept	13.589	0.8470	16.045	<0.0001
InfectY1	Rab_spread_rate	2.924	-	383.830	<0.0001
	s(Kmean)	1.991	-	1537.866	<0.0001
	s(Init_infect_y)	1.972	-	383.033	<0.0001
	s(Init_infect_x)	1.805	-	6.834	0.051
	ADL_mvt_25pc	0.241	0.0864	2.793	0.0052
	Age_Ind	0.0906	0.0061	14.813	<0.0001
	YOYF_mortality	–0.0224	0.0052	–4.332	<0.0001
	Intercept	9.116	0.3450	26.422	<0.0001
SpatMV	te(Kmean, Rab_spread_rate)	8.941	-	578.3	<0.0001
	s(Age_Ind)	2.949	-	466.8	<0.0001
	Kcv	1.195	0.0745	16.033	<0.0001
	Nb_birth_peaks	–0.138	0.0331	–4.175	<0.0001
	YOYM_motality	–0.007	0.0029	–2.457	0.0141
	Intercept	3.634	0.1213	29.960	<0.0001
